# Editorial: Non-lethal approaches in environmental science

**DOI:** 10.3389/fgene.2022.1086605

**Published:** 2022-11-24

**Authors:** Denina B. D. Simmons, Vince Palace

**Affiliations:** ^1^ Faculty of Science, Ontario Tech University, Oshawa, ON, Canada; ^2^ IISD Experimental Lakes Area, Winnipeg, MB, Canada

**Keywords:** non-lethal methods, wildlife, environmental monitoring, contaminants, stressors

The goal of this research topic was to bring together a collection of non-lethal methods and research that could be used when working with threatened and endangered species, or in environments where populations are already under stress due to the presence of contaminants. With advancements to genomic methods and non-invasive genomic assay techniques such as eDNA, non-targeted analyses for proteins and metabolites in biofluids, and cellular level RNAseq, drones, telemetry, and *in vitro* cell assay, we can collect more information from minimal amounts of biological samples than we could in the past. Now, more than ever, we are ready to conduct environmental monitoring and assessments without the sacrifice of animal wildlife.

In our research topic, we received five papers—one review, two method articles, and two original research articles from a globally diverse set of authors. In the literature review, Thorstensen et al. describe in detail how different methods of tagging wild fish in natural environments can be used to track movements and estimate population size. They also describe the best practices for tagging without causing stress to the fish. They then explore how physiological, isotopic, and contaminant measurements from small samples like small tissue biopsies and blood can be integrated with movement data—providing a holistic approach to studying and monitoring wild fish health and behavior.

We also gathered two method papers that showcase safe and non-lethal methods for sampling blood from Rainbow trout (Pollard et al.) and using blood and feathers from Tawny owl nestlings to conduct *in vitro* primary cell assays (Kroglund et al.). From fish (Pollard et al.), four different blood drawing post-treatments were tested and then blood plasma was prepared as either fresh plasma, air-dried plasma, or dried plasma from a special plasma spot filtration card device—where the latter two methods would be easier to prepare and store under field conditions. These different plasma preparation techniques were compared after measuring proteins and metabolites in each set of samples—to determine which sample preparation method could provide the most information. In owl nestlings (Kroglund et al.), feather fibroblast cells and peripheral blood mononuclear cells were isolated and cultured from the pulp of secondary wing feathers and whole blood, respectively. These types of feathers and the blood cells can be collected non-lethally, and with secondary feathers, non-invasively. The cells continued to proliferate and were viable up to 30 days after being added to 96 well plates. This assay offers massive potential to study immune and toxicological responses to chemicals and stressors in raptor species, including Tawny Owls, and could be applied to study valuable endangered bird and raptor species.

Finally, we included two research papers that demonstrated the use of non-lethal methods to study real wildlife populations: 1) one study that measured trace metal levels in faeces that came from different animals in African Savannahs that occupy different trophic levels (Webster et al.), and 2) a study that conducted RNA sequencing on epidermal mucous samples collected from Lake Charr exposed to diluted bitumen within a boreal lake (Andrzejczyk et al.). These studies demonstrate how we can understand interactions of contaminants and stressors with animals in their natural habitats without causing harm to precious wildlife in contaminant impacted environments and delicate ecosystems that are threatened by climate change and habitat loss.

